# Using Entropy in Web Usage Data Preprocessing

**DOI:** 10.3390/e20010067

**Published:** 2018-01-22

**Authors:** Michal Munk, Lubomir Benko

**Affiliations:** 1 Department of Informatics, Constantine the Philosopher University in Nitra, Tr. A. Hlinku 1, 949 74 Nitra, Slovakia; 2 Institute of System Engineering and Informatics, University of Pardubice, Studentska 95, 532 10 Pardubice, Czech Republic

**Keywords:** data preprocessing, information entropy, web usage mining, session identification, Reference Length

## Abstract

The paper is focused on an examination of the use of entropy in the field of web usage mining. Entropy creates an alternative possibility of determining the ratio of auxiliary pages in the session identification using the Reference Length method. The experiment was conducted on two different web portals. The first log file was obtained from a course of virtual learning environment web portal. The second log file was received from the web portal with anonymous access. A comparison of the results of entropy estimation of the ratio of auxiliary pages and a sitemap estimation of the ratio of auxiliary pages showed that in the case of sitemap abundance, entropy could be a full-valued substitution for the estimate of the ratio of auxiliary pages.

## 1. Introduction

Analysis of the behavior of web users is greatly influenced by the preprocessing phase of web usage mining. Many authors offered many solutions and various methods for log files preprocessing. Log files are the corner-stone of analysis of what the users have done on the web portal. The main steps of the web usage data preprocessing are data cleaning, web user identification, session identification, and path completion [1,2]. Each of the phases greatly influences the final results of the analysis. This paper deals with the improvement of data preprocessing of web usage data. It is focused on one particular method of session identification and introduces a novel approach to its usage with the help of entropy. Information theory and entropy were first introduced by C. Shannon [3], and had been used in different fields of informatics. Entropy is used as a measure of disorder, where lower entropy means order, and on the other hand, higher entropy means disorder. Following Shannon’s definition [3], entropy can be used as a measure of uncertainty in a data set. It was used as a starting point in creating a novel approach to estimate the ratio of auxiliary pages, which is an important parameter for session identification using the Reference Length method. 

The rest of the paper is structured as follows: in Section 2, entropy is presented and the related work of other authors about entropy is summarized. The theoretical and research background is described in Section 3. Section 3.1 (Theoretical Background) describes the Reference Length method of session identification. Section 3.2 (Research Background) contains the experiments’ results focusing on the influence of sitemap estimation of the ratio of auxiliary pages to the accuracy of the session identification, especially for Virtual Learning Environment (VLE) portal and portal with anonymous access. Section 4 deals with a description of the introduced approach, methodology, and results of the experiment. Subsequently, the discussion is offered in the last section.

## 2. Related Work

Entropy comes from the field of thermodynamics [4], and it was used to provide a statement of the second law of thermodynamics on the irreversibility of evolution. It was understood that an isolated system could not pass from a state of a higher entropy to a state of a lower entropy [5]. Entropy was firstly mentioned in the field of information theory by C. Shannon. He used a thought experiment to propose a measure of uncertainty in a discrete distribution [5]. The definition of entropy in information theory is as a degree of disorder or randomness in the system. Based on Shannon’s definition in [3,6,7], given a random variable class *C* with a discrete probability distribution

(1) $${\left\{p_{i}=Pr\left[C=c_{i}\right]\right\}}_{i=1}^{k},\sum_{i=1}^{k}p_{i}=1,$$

where $c_{i}$ is the *i*th class, then entropy H(C) is defined as

(2) $$H\left(C\right)=-\sum_{i=1}^{k}p_{i}\log p_{i},$$

while the function decreases from infinity to zero and $p_{i}$ takes values from in the range <0, 1> [3,6]. Entropy as a modeling tool was formulated in [8], and it is known as Maximum entropy [9,10]. 

Other authors have partially used entropy in the field of web usage mining. Kumar et al. [11] implemented an algorithm of semantic-synaptic web mining algorithm that is based on the entropy value and information content. The algorithm deals with clustering of the web page and after that the entropy of web pages is calculated. The algorithm was examined on a large data set of web pages and the results indicate that the web pages with low entropy value generally provide the most relevant data. Liu et al. [12] presented a novel approach to feature selection based on the quality of information. Authors used the maximum-nearest neighbor to generalize Shannon’s information theory. The proposed algorithm was verified on a set of datasets of UCI Repository of machine learning databases. The authors compared their algorithm with other popular feature selection of algorithms. The results showed that the proposed approach is more effective than other feature selection of algorithms. Arce et al. [13] presented a heuristic approach based on simulation annealing for the problem of sessionization. The sessionization problem addresses reconstruction of the user sessions. The quality of reconstruction is measured with respect to the power law for the size of the sessions on a web site. Entropy was used to identify the interesting partitions of the log file of a web portal. The authors measured the diversity of a given IP address regarding the entropy of the IP address. Levene and Loizou [14] introduced a novel algorithm for computing the entropy of Markov chain that represents the trail of web navigated pages by a user or a group of users. This allows for the authors to compute the probability of a typical trail, which can be also used to personalize ranking algorithms. The way the algorithm computes the entropy relates with the way users surf the web and how the web log data is collected. Authors also presented an extension of the algorithm to deal with high-order Markov chains of bounded order. Maung and Win [15,16] introduced a new heuristic to test suite reduction by applying entropy gain theory. The use of a large number of test cases from web usage logs is not practical within a time constraint. The algorithm combines the user session data and structural analysis of the examined web site to generate the test suite. The entropy-based reduction method is used to test case reduction for user session based testing. Despite the good empirical results, authors did not compare their approach to current user session based testing techniques. Jin et al. [17] combined the maximum entropy model for the recommendation system. Their results showed that the recommendation system could achieve better accuracy, than a standard Markov model for page recommendation. It was also showed a better interpretation of web users’ navigational behavior. Wang et al. [18] proposed an unified minimax entropy approach to user preference modeling with multidimensional knowledge. Authors used maximum entropy model to learn the check-in preferences of users. Check-in preference is an important component of Point-of-Interest prediction and recommendation. The proposed minimax entropy model is used to estimate the parameters with the preference learning. Ibl and Capek [19,20] used level of uncertainty (entropy) as an indicator for determining the degree of predictability of modelled systems. The authors focused on measuring the uncertainty of a process model that was modelled using stochastic Petri nets. Hui et al. [21] focused on the comparison of the performance of maximum entropy with the Naïve Bayes and Support Vector Machine algorithms, where entropy outperformed all of them. The algorithms were evaluated based on accuracy. The only downside of maximum entropy was its slow running when compared to other algorithms. Erlandsson et al. [22], in their article, showed how association rule learning could be used to predict user participation on social media pages. Data used for the experiment was gathered from Facebook. The results showed that using association rule learning, it is possible to identify influential users and can predict user participation in social media pages. Berezinski et al. [23], dealt with one task of data mining—anomaly detection. The authors presented an alternative entropy-based approach to anomaly detection caused by botnet-like malware. Jozani and Ahmadi [24] explored the ranked set sampling that has many applications in various fields. They have considered the information content of perfect and imperfect ranked set sampling data using the Shannon entropy, Rényi and Kullback-Leibler information measures. The results of their experiments showed desirable properties of ranked set sampling in comparison to commonly used simple random sampling in the context of information theory. Authors in [25,26] have focused on the problem of mining the structure of web site consisting of many hyperlink documents. The authors proposed an entropy-based analysis to analyze the entropy of anchor texts and links. Kao et al. [26] employed the entropy information to analyze the information measures of article sets. Entropy is used also to analyze the behavior on modern online social platforms. Wei and Zhu [27] introduced a cascade detection mechanism based of a web spam on entropy-based outlier mining algorithm. The entropy-based outlier mining algorithm consists of two steps: data discretizing and samples grouping to different sets, both based on entropy. Primarily, Agreste et al. [28] focused on analysis of user behavior on social platform were they created three profiles for each user and compared the profiles between each other using entropy and mutual information. De Meo et al. [29] analyzed the correlation between social and tagging behavior of the users on different social sharing systems.

The proposed work differs from the above works since it is oriented on a specific method of session identification, namely the Reference Length method. The aim of this research is to find an alternative estimation of the ratio of auxiliary pages used in Reference Length method. 

## 3. Methods

This section describes the theoretical and research backgrounds that inspired our experimental direction using the entropy. The theoretical background provides necessary information about the process of session identification and one of its methods—Reference Length—is important for presented research. The research background deals with two previous experiments that served for this research as a starting point in discovery of the entropy-based estimation of a key parameter of the session identification method Reference Length. Experiments are focused on the sitemap estimation of the ratio of auxiliary pages to the accuracy of the session identification especially in the VLE and on portal with anonymous access.

### 3.1. Theoretical Background

In the process of session identification, it is important to divide the user’s visits into sessions. The session is characterized by an activity of one user in a certain time on the web portal [30]. Issues with session identification can be solved by time-oriented heuristics, structure-oriented heuristics, and navigation-oriented user session identification. Time-oriented heuristics *h1* and *h2* create sessions based on a time window, for example, 30 or 10 min [1,31]. Structure-oriented heuristic, such as *h*-*ref*, identifies new sessions based on another parameter, such as a field referrer, where if the URL is not followed by the referrer, it becomes a new session [1,31,32]. Navigation-oriented methods assume that two sets of transactions (auxiliary-content and content-only) can be formed. In experiment [33], based on the sitemap the assumption about the ratio of auxiliary pages estimated for the session identification using the Reference Length method, was made. The Reference Length method falls into this category. It is based on the assumption that the amount of time, that the user spends on a page depends on whether the page is classified as an auxiliary or content page [1,32,33,34,35].

**Figure 1:**
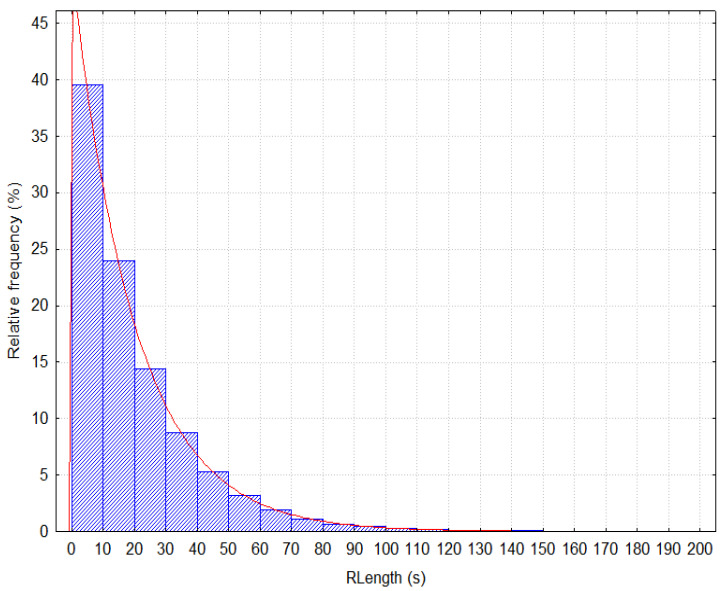
Distribution of the variable *RLength*.

Figure 1 shows a histogram depicting the distribution of the variable *RLength* representing the time spent on the pages of a particular portal. We assume that the variance of times spent on the auxiliary pages is small because the user “only” passes through the pages to his/her search target. The auxiliary page shapes the left part of the graph. The length of the time spent on content pages has a higher variance and shapes the right part of the graph. 

Provided that, the variable *RLength* has an exponential distribution (*Chi-square* = 21.40632; *p* = 0.06527) and the assumption about the portion of auxiliary pages is made (0≤p<1), we can determine the cut-off time

(3) $$F^{-1}\left(p,\lambda \right)=C=\frac{-\ln\left(1-p\right)}{\lambda },$$

separating the auxiliary pages from the content ones [33]. The maximum likelihood estimation of the parameter λ is

(4) $$\hat{\lambda }=\frac{1}{\bar{RLength}},$$

where $\bar{RLength}$ is the observed mean of times spent on the pages.

If we have an estimation of the cut-off time C, then the session (visit) is a sequence *k* of the visited pages with the time mark, for which the following is valid: the first k−1 pages are classified as the auxiliary pages. The time spent on these pages is less or equal to the cut-off time and the last *k*th page is classified as a content one. The time that was spent on this page is higher than the cut-off time. 

**Figure 2:**
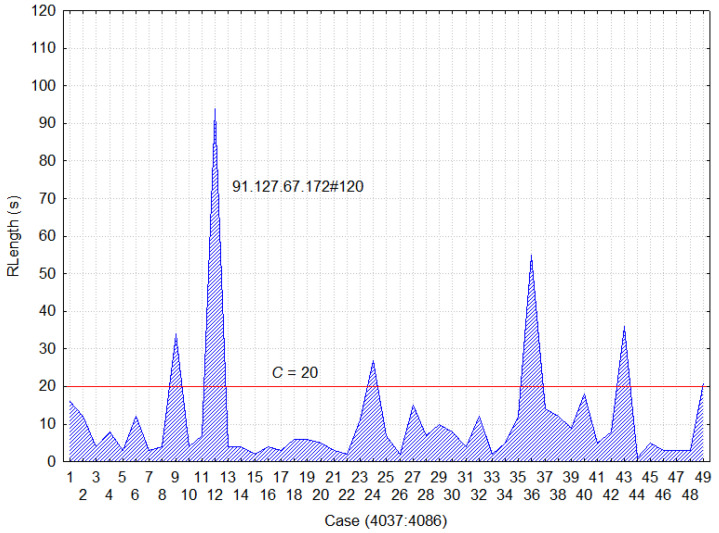
Reference Length method.

Each session ends with a content page as illustrated in Figure 2. The *x*-axis represents a sequence of the visited pages from the given IP address and agent (91.127.67.172#120) ordered according to access time. The *y*-axis depicts the time spent on the page and the cut-off time estimation is 20 s. The first session consists of pages (Figure 2) with sequence numbers from 1 to 9, the first 8 are classified as auxiliary pages, and the 9th is classified as a content one, whereby the next session is a formation of pages with sequence numbers from 10 to 12.

### 3.2. Research Background

Two previous experiments [33,35] are closely related to the aim of this paper. We focused on the influence of estimation of the ratio of auxiliary pages to the accuracy of the session identification. These experiments provide the reader with a solid review of the issues that are related to the estimate of the ratio of auxiliary pages in the process of session identification in the context of Web Usage Mining. Both experiments [33,35] dealt with the analysis of actionable (useful), trivial and inexplicable sequence rules [36]. 

**Figure 3:**
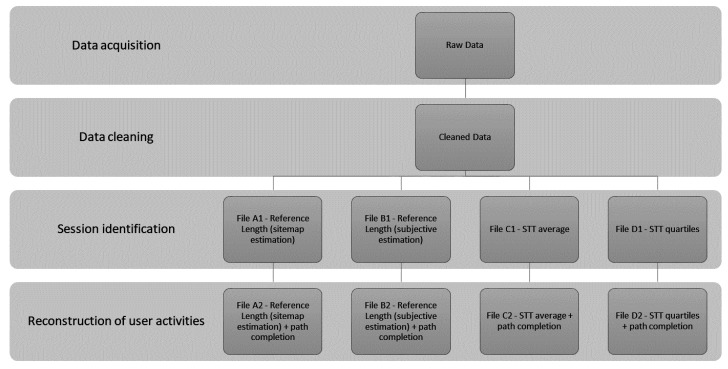
Application of data preparation to the log file of Virtual Learning Environment (VLE).

The first experiment [35] dealt with the analysis of several data preprocessing techniques for session identification and path completion. Authors compared eight files in different stages of data preprocessing (Figure 3). Using the STATISTICA Sequence, Association, and Link Analysis were extracted sequence rules from frequent sequences for each examined file (Figure 3). Authors expected that the identification of sessions using the Reference Length method, estimated from a sitemap would have a significant impact on the quantity of extracted rules. This was not proved as session identification using the Reference Length method based on sitemap did not have an impact on the quantity of extracted rules in case of files without and with path completion. On the other hand, an assumption concerning the impact of the method based on sitemap on increasing the portion of useful rules was proved. It was proved that the use of the Reference Length method with the estimate of the ratio of auxiliary pages from the sitemap, has significantly increased the number of useful sequence rules found.

**Figure 4:**
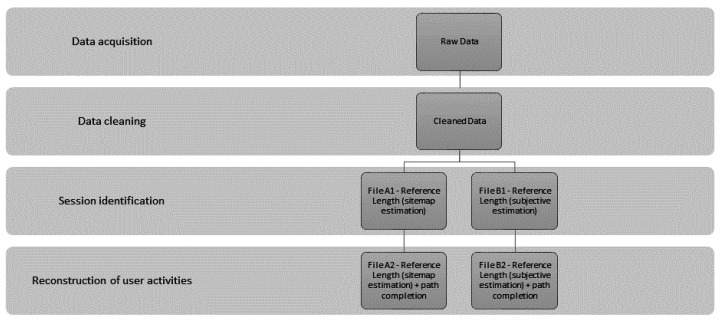
Application of data preparation to the log file of web portal.

The second research [33] dealt with the similar issue of the estimate of the ratio of auxiliary pages and its impact on the preprocessing phase. The log file of a university website was used to compare the methods of the estimate of the ratio of auxiliary pages. Using sequence rule analysis (STATISTICA Sequence, Association, and Link Analysis), sequence rules were extracted from frequent sequences with the minimum support 0.01 for each examined file (Figure 4). The most rules were found in files with path completion, over 79% in case of the file with the estimate of the ratio of auxiliary pages from the sitemap (File A2), and 98% in case of the file with the ratio based on a subjective estimate (File B2). The value of Kendall’s coefficient of concordance (approximately 0.37) confirmed the *Q* test results (*Q* = 86.63190; *df* = 3; *p* < 0.001) and based on these results, the zero hypothesis which reasons that the incidence of rules does not depend on individual levels of data preparation, is rejected at the 1% significance level.

A closer look at the results (Table 1) shows that the files without path completion (Files A1 and B1) contain almost identical rules, except four rules (5%) for the file with the subjective estimation of the ratio of auxiliary pages (B1). In case of files with path completion (Table 2) was proved a statistically significant difference in 16 new rules (almost 21%) in favor of file with subjective estimation (B2).

Based on the contingency coefficients (Coef. C, Cramér’s V), which represent the degree of dependency between two nominal variables, there is a moderate dependency among the portion of useful, trivial, and inexplicable rules, and their occurrence in the set of discovered rules of files without path completion (A1: 0.40, B1: 0.37) separately. Besides, the contingency coefficient is statistically significant. The obtained results for the files with path completion (A2, B2) were more interesting. A moderate dependency (0.30) was found among the portion of useful, trivial, and inexplicable rules, and their occurrence in the file A2 (Table 3). The coefficient value for the incidence of rules and types of rules for the file B2 was approximately 0.11, where 1 represents perfect dependency and 0 means independence, which means that there is only a small dependency for the file B2 (Table 4). Also, the contingency coefficient is not statistically significant (Table 4). The file B2 contained the most inexplicable rules, but the portion of useful rules was the same for all of the files.

The results of the sequence rule analysis were examined not only for the quantity of extracted rules, but also for the quality [33]. Quality of rules was examined by two indicators: support and confidence [36]. Statistically significant differences were proved among file A1, A2, B2, and between files B1, B2 regarding the average support of the found rules (Table 5). In terms of the average confidence of found rules, there were identified statistically significant differences between files A1, A2, and between files A1, B2 (Table 6). The impact on the quality and quantity of extracted rules was proven only after path completion. On the contrary, path completion is dependent on the accuracy of session identification.

The results of the experiments [33,35] showed that the ratio of auxiliary pages has an impact on the quantity of found rules only in the case of files with path completion. There was proved no impact on the increase of useful rules, but inappropriate estimation of the ratio of auxiliary pages can lead to an increase of trivial and inexplicable rules. Based on the results it was recommended to use the estimation of the ratio of auxiliary pages from the sitemap. The downside of this solution could be the abundance of sitemaps or the abundance of current sitemaps because web portals are always changing. It inspired this papers’ experiment to find a solution that would not need the sitemap, and would offer similar results to estimate the ratio of auxiliary pages as the sitemap estimate of the ratio of auxiliary pages.

## 4. Experiment

The sitemap can offer an accurate estimate of the ratio of auxiliary pages. This could be complicated if the examined web portal was changed in the meantime and the sitemap becomes inaccurate or incomplete. Another issue would be a missing sitemap. That means that the sitemap estimate of the ratio of auxiliary pages cannot be used for the Reference Length method of session identification. One option would be to extract the sitemap from the log file, but this would only result in obtaining an incomplete sitemap. An alternative could be offered by the use of entropy as a measure of uncertainty to estimate the ratio of the auxiliary pages from the log file.

### 4.1. Methodology

The log files used in the experiment were extracted from the VLE portal and web portal with anonymous access. Research methodology proceed from the results of the above research articles [33,35]. The experiment is comprised of the following steps:

1. Data acquisition—obtaining the log file and sitemap and defining the observed variables in the log file to obtain the necessary data (IP address, date and time of access, URL address, etc.).
2. Data cleaning—removing unnecessary data, such as access to images, styles, etc., and removing the accesses of robots of search engines.
3. User identification—based on *IP address* and *UserAgent*.
4. Data transformation and sequence identification—creating time variable *UnixTim*e from date and time of access and creating variable *Length* based on time window that the user would most likely spend on a web portal.
5. Creating a data matrix from the log file with unique web portal pages and corresponding time spent on the page by the users.
6. Calculating *Relative Mean Time* spent by the users for each web portal page from the *Length* variable.
7. Calculating *Entropy* from *Relative Mean Time* for each web portal page and *Average Entropy* and *Quartiles Entropy* for the whole web portal.
8. Estimate the ratio of auxiliary pages based on entropy—dividing the web portal pages to auxiliary and content based on the entropy and *Average*/*Quartiles Entropy*.
9. Estimate the ratio of auxiliary pages from the sitemap—the sitemap consists of the variables *URL* and *Referrer*, where the number of auxiliary pages corresponds to the number of unique referring pages in the used web portal.
10. Draw a comparison of the results of ratio of the auxiliary pages estimation by various techniques.

The experiment expectations are in finding an alternative method to estimate the ratio of auxiliary pages in case the sitemap is missing or is inaccurate.

### 4.2. Results

The experiment was conducted separately for log files of two different web portals. The first log file was received from the virtual learning environment portal. This type of portal represents web portals with the need of users’ login and cannot be accessed anonymously. The second log file represents a web portal with anonymous access, in our case, it is our university portal. 

Both log files were preprocessed using standard web usage data preprocessing techniques, as in [33,34,37]. The preprocessed log files were imported into the database separately. Several calculations, involving entropy, were conducted with the aim to find a way to distinguish auxiliary pages from content pages based on the length of time, which the user spent on each site. It resulted in the creation of an algorithm that could be able to calculate entropy for a specific page based on a random variable *RLength*, representing the length of the time spent on each web page of the portal. With the use of the algorithm, the variable *Relative Mean Time* was created, representing the time which the user spent on the page. From the variable *Relative Mean Time*, *Entropy* was derived from the individual page, and a new data matrix was created (Table 7) containing the *Entropy* of each page. Subsequently, it was calculated the average length of all accesses on the web portal, and it served as the cut-off value of time that divides the pages into auxiliary and content pages. The reason why calculated entropy was higher than 1 was a greater number of examined categories. All of the pages with a higher *Entropy* than the *Average Entropy* ($Entropy_{Mean}$) of the whole portal, will be classified as auxiliary pages. On the other hand, pages with a smaller *Entropy* than *Average Entropy*, will be classified as content pages. Quartiles are another option for specifying the threshold time, spent on the web portal. The time was calculated by

(5) $$Entropy_{Quartiles}=Q_{III}+1.5Q,$$

where $Q_{III}$ represents the upper quartile and Q represents the quartile range. This process was done separately for each of the log files.

**Figure 5:**
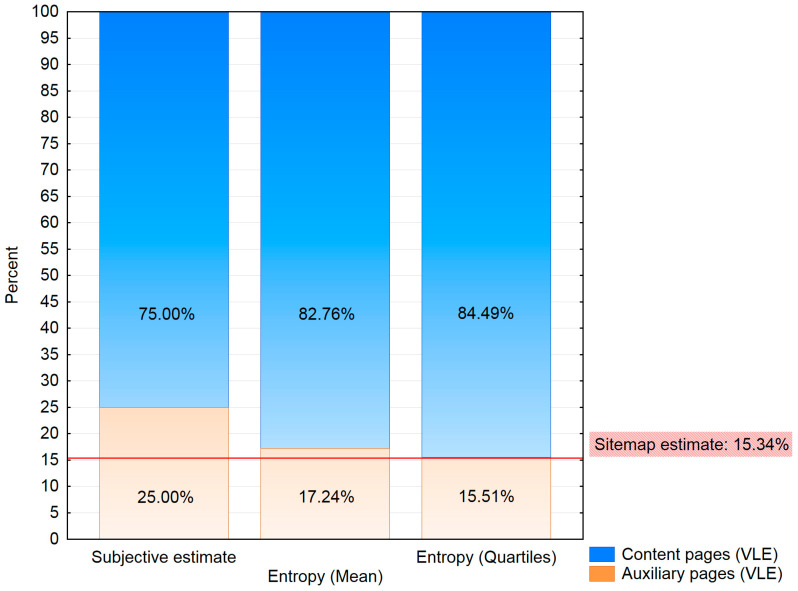
Ratio of auxiliary and content pages based on different estimates of the ratio of auxiliary pages for VLE web portal.

The stacked Plot (Figure 5) visualizes the reached ratios of content and auxiliary pages examined by estimation methods to estimate the ratio of auxiliary pages for the log file of the VLE portal. In the log file of the VLE portal, 58 pages were identified. Using the algorithm, ten pages were classified as auxiliary pages, and the rest (48 pages) were classified as content pages. Therefore, the ratio of auxiliary pages of the VLE portal was 17.24% (Figure 5). Using quartiles, nine pages were classified as auxiliary pages, and 49 pages were identified as content pages. As the results of previous studies [33,37] showed, the ratio estimated from the sitemap offered the best results for using the Reference Length method as session identification. Based on that, the aim of this experiment was to find a solution to estimate the ratio of auxiliary pages from the log file without using the sitemap. The results (Figure 5) for the VLE portal showed that the *Quartiles Entropy* ($Entropy_{Quartiles}$) was almost similar (15.51%) to the sitemap estimate of the ratio of auxiliary pages (15.34%). The *Average Entropy* (17.24%) was little higher than the sitemap estimate of the ratio of auxiliary pages (15.34%), but still offers more accurate results than the subjective estimate of the ratio of auxiliary pages (25%).

**Figure 6:**
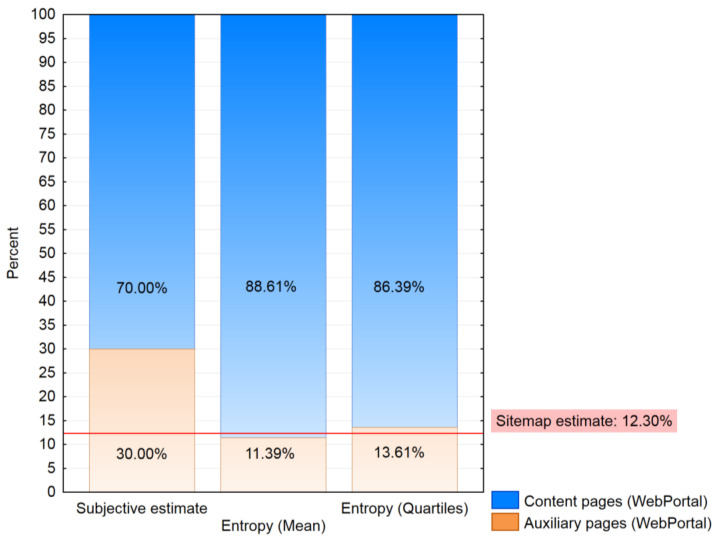
Ratio of auxiliary and content pages based on different estimates of the ratio of auxiliary pages for web portal with anonymous access.

The process of calculating entropy was repeated for the log file of a web portal with anonymous access. In this case, the difference between the subjective (30%) and sitemap (12.30%) estimate of the ratio of auxiliary pages was much higher than in the case of a previous web portal (Figure 6). The results of previous studies [33,34] showed that the sitemap estimate of the ratio of auxiliary pages offers better results regarding the quantity and quality of extracted rules. The results of the entropy estimate of the ratio of auxiliary pages (Figure 6) showed a similar behavior for the web portal with anonymous access and the VLE web portal both (Figure 5). The web portal with anonymous access contained 1764 pages. In this case, it could be more appropriate to use the *Average Entropy* (11.39%). However, the results of *Quartiles Entropy* (13.61%) were close to the sitemap estimate of the ratio of auxiliary pages (12.30%). The selection of the appropriate method of entropy estimate of the ratio of auxiliary pages could be in this case based on the decision of the web portal analyst. The choice of *Average Entropy* would prove to be generating stricter rules and could extract less rules than the *Quartiles Entropy*. The recommendation of *Quartiles Entropy* would be more appropriate because there is no a big difference of the estimate of the ratio of auxiliary pages compared to the sitemap. Also, for the first web portal, the results of *Quartiles Entropy* were more accurate (Figure 5). Apart from the need of selecting the more appropriate method, it can be said that the results of both methods offer similar results as the sitemap estimate of the ratio of auxiliary pages.

## 5. Discussion

Session identification can be carried out using various methods, but the Reference Length method offers good results. The results of the above researches [33,35] showed the good results of using sitemap estimation of the ratio of auxiliary pages. The results of [33] showed that the session identification using the method Reference Length with sitemap estimation of the ratio of auxiliary pages has an impact on the quantity and quality of extracted sequence rules after path completion. An inappropriate estimation of the ratio of auxiliary pages may increase the number of extracted rules, but mainly trivial and inexplicable rules. In case of the VLE, the results of [35] have shown similar results. The least inexplicable rules were found in case of the file prepared by the Reference Length method with sitemap estimation of the ratio of auxiliary pages. It was also shown that the most useful sequence rules were extracted using this method. This article presented an extension of the Reference Length method proposing an alternative way to estimate the ratio of auxiliary pages. The entropy estimation of the ratio of auxiliary pages is a good option when the problem with the sitemap of the examined web portal exists. The downside of the sitemap is its lack of accuracy in terms of constant web changes. This can result in the estimate distorting of the ratio of auxiliary pages in the sitemap. A more serious problem would be a missing sitemap where the use of session identification method Reference Length with sitemap estimate of the ratio of auxiliary pages would not be possible. The only option would be to relate to the subjective estimation of the ratio of auxiliary pages or use different session identification method. As it was shown in the above experiments [33,35], a subjective estimate is not as accurate, in terms of the ratio of auxiliary pages as a sitemap estimate.

**Figure 7:**
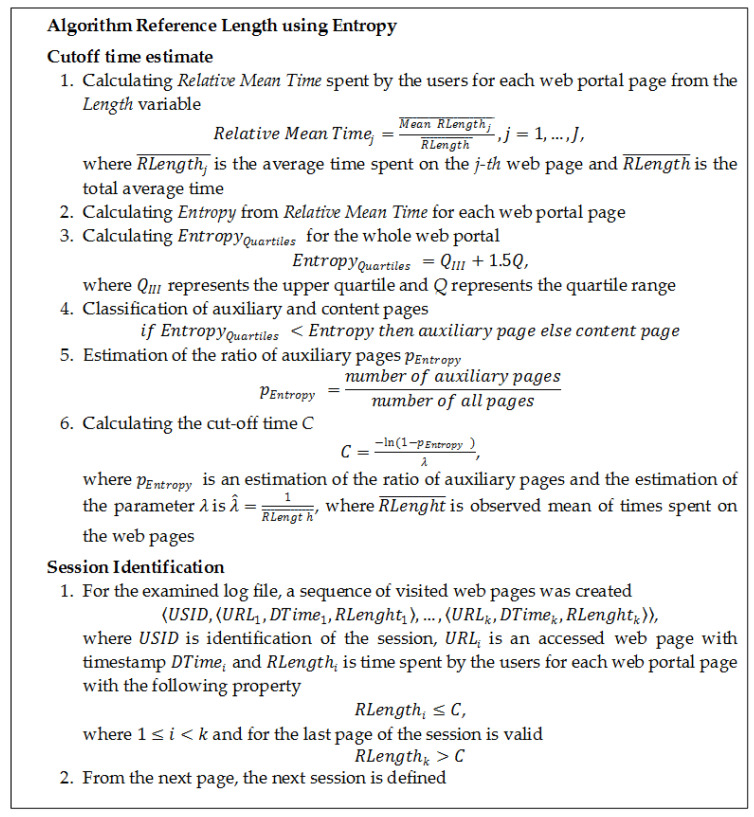
Algorithm of Reference Length method using Entropy.

This paper introduced entropy as an alternative to estimate the ratio of auxiliary pages in session identification using Reference Length method. Other authors [11,13,14,15,16,17] used entropy in the field of web usage mining, but as it consists of many areas and phases of the process of web usage analysis. This paper focused on a specific phase of the data preparation—session identification method Reference Length. In this paper, a new method of how to estimate the ratio of auxiliary pages using entropy was introduced (Figure 7). The method uses entropy as a tool to estimate the ratio of auxiliary pages from the time spent on the web pages of web portal. As this method works directly with the examined log file, the results should correspond more accurately with the structure of examined web portal at the time of obtaining the log file. This could be beneficial in the case of historical data and web portals for which sitemaps could not be created or are incomplete. The experiment was carried out for two different log files obtained from two different web portals. Firstly, the experiment was done on a log file from a VLE portal. The log file was extracted for a specific course and offered a good starting point for the experiment because it contained fewer records than log files from bigger web portals. Also, the advantage of using this type of web portal was that there was no need to clean the log file from unnecessary data. After finding the solution to the problem of estimating the ratio of auxiliary pages without a sitemap, the experiment was repeated on a log file of a web portal with anonymous access. The log file of the web portal with anonymous access contained more records, and the web portal was also more complex. In the case of the VLE portal, entropy estimation of the ratio of auxiliary pages was almost similar to the sitemap estimation of the ratio of auxiliary pages. The results of the entropy estimation of the ratio of auxiliary pages for the web portal with anonymous access were also similar to the sitemap estimation of the ratio of auxiliary pages. The aim of this research was to find an alternative estimation of the ratio of auxiliary pages. Results of the ratio of auxiliary pages for both web portals showed that entropy offered similar results to estimate the ratio of auxiliary pages as the sitemap estimate presented in [33,35].

Future work on using entropy in the field of Web Usage Mining could be in a determination of the size of the content page. Based on data in the log file, entropy could be used as a measure of content-rich pages. It could be assumed that a higher value of entropy would indicate a higher proportion of content on a particular page of the web portal. It could serve as an alert for the web portal administrator to review such content-rich pages. This could indicate that the web page visitor has problems to understand the content or that the page contains too much information. The solution for the web portal administrator could be to divide the concerned web page into more categories. The idea is based on a similar research [38,39], in which the page rank for each page of the web portal was calculated. 

## Figures and Tables

**Table 1:** Crosstabulations of File A1 × File B1.

A1\B1	0	1	∑
**0**	39	4	43
	50.00%	5.13%	55.13%
**1**	0	35	35
	0.00%	44.87%	44.87%
**∑**	39	39	78
	50.00%	50.00%	100.00%
**McNemar (B/C)**	*Chi-square* = 2.25000; *df* = 1; *p* = 0.134		

**Table 2:** Crosstabulations of File A2 × File B2.

A2\B2	0	1	∑
**0**	0	16	16
	0.00%	20.51%	20.51%
**1**	1	61	62
	1.28%	78.21%	79.49%
**∑**	1	77	78
	1.28%	98.72%	100.00%
**McNemar (B/C)**	*Chi-square* = 11.52941; *df* = 1; *p* = 0.00069		

**Table 3:** Crosstabulations: Incidence of rules × Types of rules: File A2.

A2\Type	Useful	Trivial	Inexplicable
**0**	0	6	10
	0.00%	14.63%	37.04%
**1**	10	35	17
	100.00%	85.37%	62.96%
**∑**	10	41	27
	100%	100%	100%
**Pearson**	*Chi-square* = 7.97115; *df* = 2; *p* = 0.019		
**Con. Coef. C**	0.30450		
**Cramér’s V**	0.31968		

**Table 4:** Crosstabulations: Incidence of rules × Types of rules: File B2.

B2\Type	Useful	Trivial	Inexplicable
**0**	0	1	0
	0.00%	2.44%	0.00%
**1**	10	40	27
	100.00%	97.56%	100.00%
**∑**	10	41	27
	100%	100%	100%
**Pearson**	*Chi-square* = 0.91416; *df* = 2; *p* = 0.633		
**Con. Coef. C**	0.10763		
**Cramér’s V**	0.10826		

**Table 5:** Homogeneous groups (not significant differences) for support of extracted rules.

File	Support Mean	1	2	3
**A1**	3.425	****		
**B1**	3.941	****	****	
**A2**	4.163		****	
**B2**	4.747			****
**Kendall Coefficient of Concordance**		0.63692		

**Table 6:** Homogeneous groups (not significant differences) for confidence of extracted rules.

File	Confidence Mean	1	2
**A1**	15.942		****
**B1**	17.123	****	****
**A2**	22.320	****	
**B2**	23.442	****	
**Kendall Coefficient of Concordance**		0.49568	

**Table 7:** Data matrix of log file of web portal extended by entropy.

*URL*	*Mean RLength*	*Relative Mean Time*	*Entropy*
/help-desk	39.6471	0.712336	0.241628
/information	52.8175	0.948967	0.049708
/conference	15.5	0.278487	0.356013
/faculties	22.1082	0.397216	0.36674
⋮	⋮	⋮	⋮
